# Effects of Nitrogen Application at Different Levels by a Sprinkler Fertigation System on Crop Growth and Nitrogen-Use Efficiency of Winter Wheat in the North China Plain

**DOI:** 10.3390/plants13121714

**Published:** 2024-06-20

**Authors:** Keke Wang, Haijun Liu, Zhuangzhuang Gao

**Affiliations:** Beijing Key Laboratory of Urban Hydrological Cycle and Sponge City Technology, College of Water Sciences, Beijing Normal University, Beijing 100875, China; wangkeke2323@163.com (K.W.); bnugaozz@mail.bnu.edu.cn (Z.G.)

**Keywords:** winter wheat, nitrogen application rates, nitrogen adsorption curve, water and fertilizer use efficiency

## Abstract

Nitrogen (N) is an essential macronutrient for crop growth; therefore, N deficit can greatly limit crop growth and production. In the North China Plain (NCP), winter wheat (*Triticum aestivum* L.) is one of the main food crops, and its yield has increased from approximately 4000 kg ha^−1^ to 6000 kg ha^−1^ in the last two decades. Determining the proper N application rates at different growth stages and in all seasons is very important for the sustainable and high production of wheat in the NCP. A field experiment with five N application rates (250, 200, 150, 100, and 40 kgN·ha^−1^, designated as N250, N200, N150, N100, and N40, respectively) was conducted during the 2017–2018 and 2018–2019 winter wheat seasons to investigate the effects of the N application rate on water- and fertilizer-utilization efficiency and on the crop growth and yield of winter wheat under sprinkler fertigation conditions. The results showed that in the N application range of 40–200 kg ha^−1^, crop yield and water- and fertilizer-use efficiencies increased as the N application rate increased; however, further increases in the N application rate (from N200 to N250) did not have additional benefits. The N uptake after regreening of winter wheat linearly increased with crop growth. Considering the wheat yield and N-use efficiency, the recommended optimal N application rate was 200 kg ha^−1^, and the best topdressing strategy was equal amounts of N applied at the regreening, jointing, and grain-filling stages. The results of this study will be useful for optimizing field N management to achieve high wheat yield production in the NCP and in regions with similar climatic and soil environment conditions.

## 1. Introduction

Winter wheat (*Triticum aestivum* L.), as one of China’s main grain crops, plays an important role in ensuring food security [1,2]. Nitrogen (N) is an important nutrient that affects wheat plant growth and yield by promoting metabolism in, and in the growth of, the roots, stems, and leaves [3]. However, excessive application of N fertilizer is common in agricultural production worldwide. This not only reduces the efficiency of N fertilizer use but also causes serious environmental problems, such as surface and groundwater pollution, increased greenhouse gas (N_2_O) emissions, and a reduction in biodiversity [4,5]. With the wheat yield increasing from 3806 kg ha^−1^ in the 2000s to approximately 5880 kg ha^−1^ at present [6,7], more N should be required to support wheat production. To optimize the N-use efficiency and enhance crop production, it is important to develop appropriate N fertilization management strategies for wheat crops in the North China Plain (NCP).

The effects of N application rates on crop growth, yield, evapotranspiration (ET), and water- and N-use efficiencies have been widely studied for different crops in various regions [8,9,10]. Generally, crops’ yields increase with increasing N application, reach peak levels at the optimal N application level, and then decrease if N is applied in excess. The optimal N application level varies among different crops and different soil environments. For wheat crops, the reported optimal N application rate varies from 180 to 230 kgN·ha^−1^ with the corresponding yield range from 7080 to 8860 kg ha^−1^ [4,11]. In the NCP, the recommended N application rate ranges from 150 to 210 kg ha^−1^ to achieve yields in the range of 7500 to 8500 kg ha^−1^. In recent decades, published reports have indicated that the wheat yield in the NCP increased from approximately 6500 kg ha^−1^ in the 2000s to 7500 kg ha^−1^ in the last 5 years, and the corresponding recommended N application rates ranged from 150–210 to 180–220 kg ha^−1^. Furthermore, the recommended N application rate (180–220 kg ha^−1^) as delivered by surface irrigation is higher than that recommended for delivery by sprinkler and drip irrigation (150–200 kg ha^−1^) because there is less N leaching using the latter two irrigation methods. Therefore, the irrigation method should be considered when optimizing the N application rate for winter wheat.

The NCP produces 58.33% of all the wheat produced in China and accounts for 51.2% of the national wheat cultivation area [7]. However, the water resources in the NCP account for only 6% of the total in China [12]. The large amount of irrigation water pumped from groundwater results in a decline in the groundwater level by approximately 1 m per year, which has caused serious environmental problems and is unsustainable for agricultural and economic development [13,14]. The sprinkler fertigation system can simultaneously supply water and nutrients to crops, which maximizes the availability of both to plants, thereby improving both water- and fertilizer-use efficiencies [15,16]. The area of land irrigated by sprinkler systems in the NCP is 3.37 million hectares at present and is expected to increase in the next 10 years because of the implementation of the “High-level Farmland Construction Project” in China. With more widespread use of the sprinkler irrigation method in the NCP, the sprinkler fertigation system will be increasingly used for wheat crops. Therefore, it is urgent to understand the responses of N uptake and growth to N application under sprinkler fertigation conditions.

Many studies have been conducted on nitrogen application rates for wheat in the NCP; however, the recommended optimal nitrogen application rates vary with regions and years [17,18,19], and the wheat yield increasing (from 5000 to 7500 kg ha^−1^) in the past 30 years needs re-evaluating these proposed N amounts when a sprinkler fertigation system is used. Therefore, the aims of this study, which was conducted over two winter wheat seasons, were as follows: (1) to explore the effects of different N application rates and splitting strategies of topdressing N on winter wheat growth, yield, and water- and fertilizer-use efficiencies; (2) to analyze the relationships between N application and the N splitting strategy in terms of their effects on wheat yield and N-use efficiency; and (3) to identify the optimal N application rate and splitting strategy to achieve high winter wheat yield and N-use efficiency.

## 2. Results

### 2.1. Meteorological Conditions

**Figure 1:**
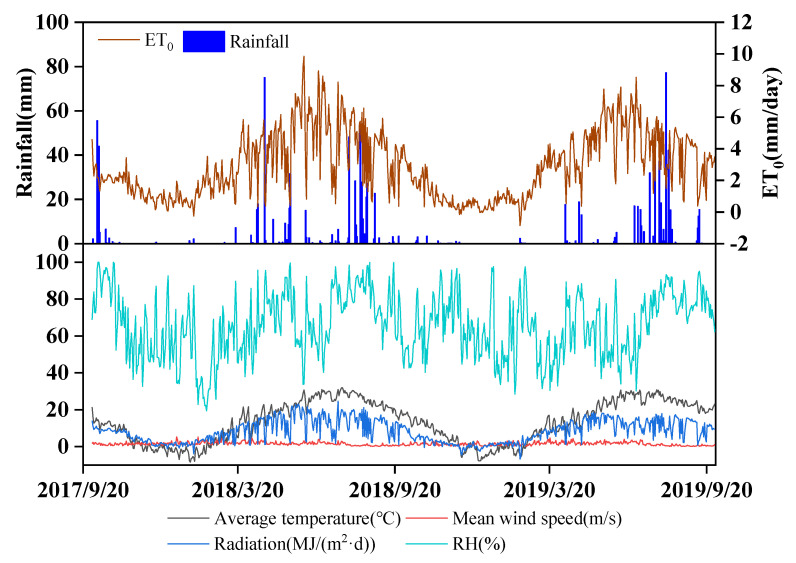
Climatic conditions from October 2017 to October 2019 at the field study site.

**Figure 2:**
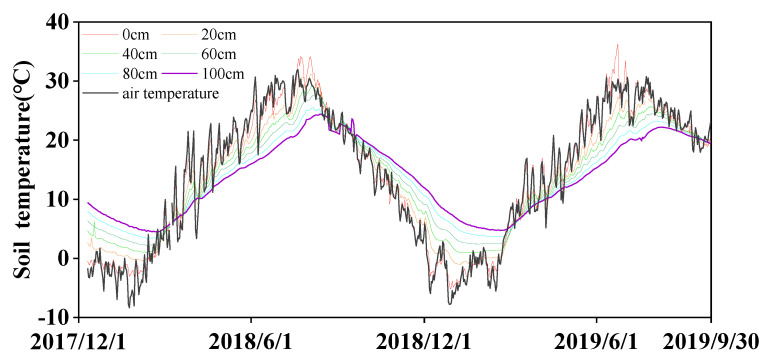
Changes in soil profile temperature from December 2017 to September 2019.

The climatic conditions from October 2017 to October 2019 are summarized in Figure 1. In the two experimental winter wheat (*Triticum aestivum* L.) growing seasons (from October to June of the following year), there were clear seasonal curves of air temperature, radiation, wind speed, and reference crop evapotranspiration (ET_o_). The seasonal rainfall amounts were 248.0 mm and 79.8 mm in the 2017–2018 and 2018–2019 wheat seasons, respectively. The seasonal ET_o_s were 606 mm and 490 mm, and high daily ET_o_ generally occurred in May with a mean of 4.91 mm·day^−1^. During winter (from November to February), the soil temperature increased with depth (Figure 2), while it decreased with soil depth from March to June. 

### 2.2. Temporal and Spatial Distribution of Soil Water Content

**Figure 3:**
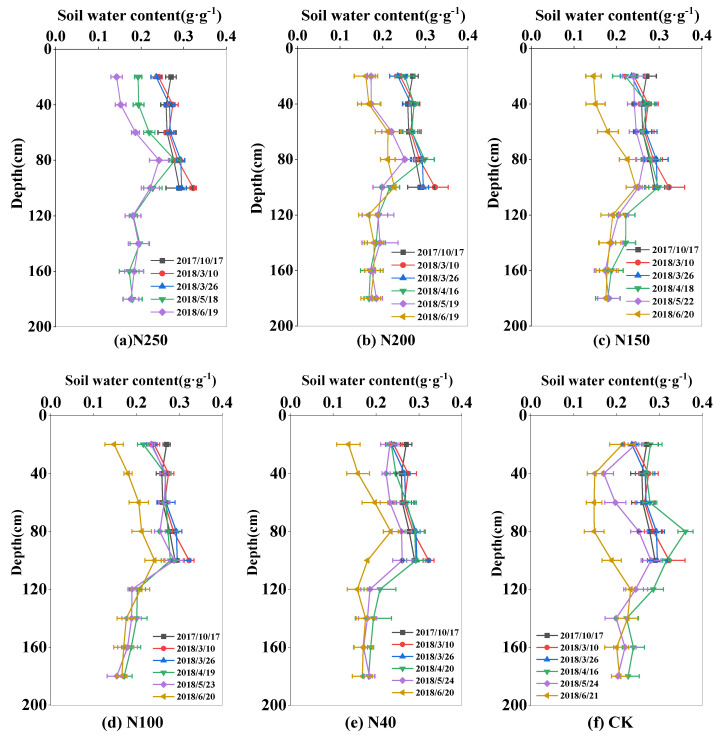
Spatial and temporal distribution of soil water content in the 0–180 cm soil layer in the 2017–2018 winter wheat growing season. Subfigs (**a**–**f**) represent N treatments of N250, N200, N150, N100, N40, and CK, respectively.

**Figure 4:**
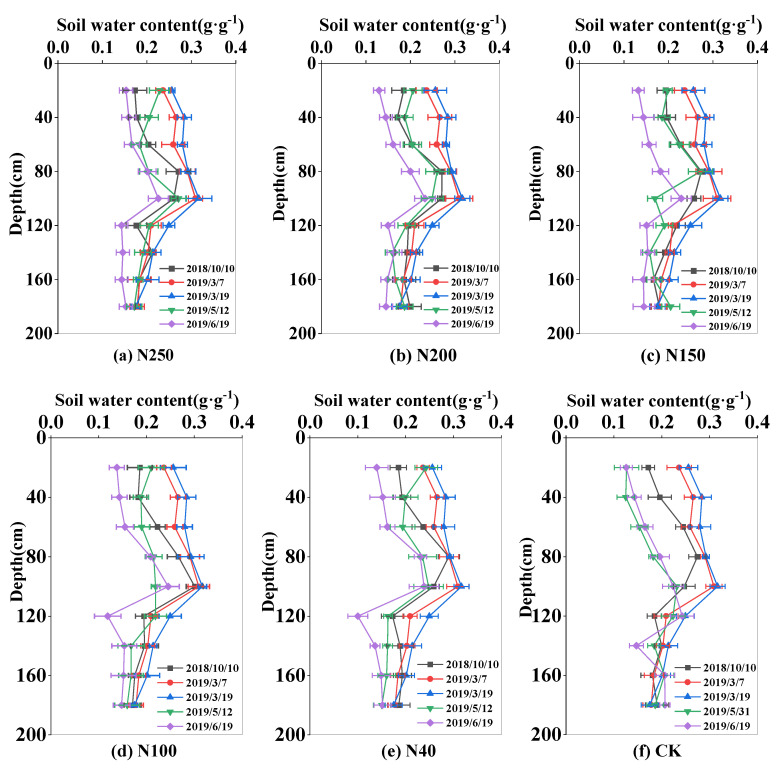
Spatial and temporal distribution in soil water content in the 0–180 cm soil layer in the 2018–2019 winter wheat growing season. Subfigs (**a**–**f**) represent N treatments of N250, N200, N150, N100, N40, and CK, respectively.

The soil water content varied greatly during the whole wheat growth season because of crop ET, rainfall, and irrigation. The soil water content was highest pre-plant (0.26–0.29 g·g^−1^) and lowest at wheat harvest (0.15–0.21 g·g^−1^). In 2017 and 2018, the soil water storage in the 0–180 cm soil layer was 562.6 mm and 580 mm in the sowing period in October of 2178 and 2018, respectively, and 424.2 mm and 401 mm in the harvest period in June of 2018 and 2019, respectively (Figure 3 and Figure 4).

Within the 0–180 cm soil layer, the soil water content showed wider variation in the 0–100 cm soil layer (0.2–0.3 g·g^−1^) than in the 100–180 cm soil layer (0.17–0.2 g·g^−1^). The larger variation in soil water content in the upper 100 cm soil layer was mainly because of water uptake by roots, and irrigation and rainfall events. However, in the CK field, the fluctuation of soil water content in the 0–100 cm was very large during the growing season, ranging from 0.15 to 0.3 g·g^−1^. At harvest, the soil water content in the 0–100 cm soil layer in the CK field was 0.17 g·g^−1^, lower than that (0.2 g·g^−1^) in the sprinkler fertigation treatments.

In the two seasons, the mean soil water contents in the 0–100 cm soil layer in the five N treatments (N250, N200, N150, N100, and N40) were 0.25, 0.25, 0.25, 0.26, and 0.25 g·g^−1^, respectively, in 2017–2018, and 0.23, 0.23, 0.23, 0.23, and 0.24 g·g^−1^, respectively, in 2018–2019. There were no significant differences (*p* > 0.05) in seasonal soil water content among the five treatments, mainly because the irrigation depth was equal among all the treatments. During most of the growth period, the soil content in the CK field was close to that in the N treatments. However, at harvest in 2018, the soil water content in the 0–100 cm soil layer was 0.2 g·g^−1^ in the CK field, which was 20% lower than that in the N treatments.

### 2.3. Temporal and Spatial Distributions of Nitrate-N Content

**Figure 5:**
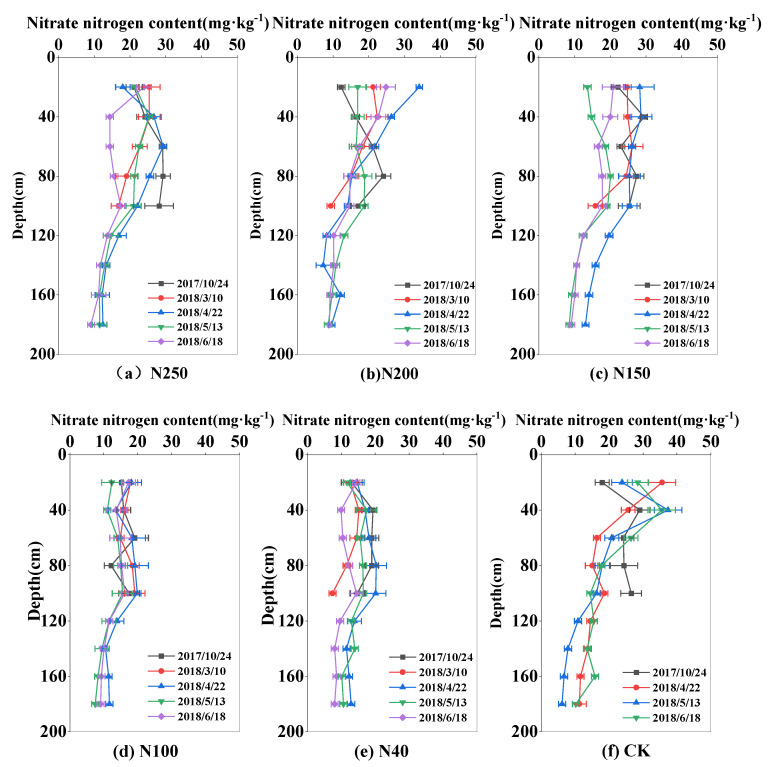
Distribution of nitrate-N content in the 0–180 cm soil layer in the 2017–2018 season. Subfigs (**a**–**f**) represent N treatments of N250, N200, N150, N100, N40, and CK, respectively.

**Figure 6:**
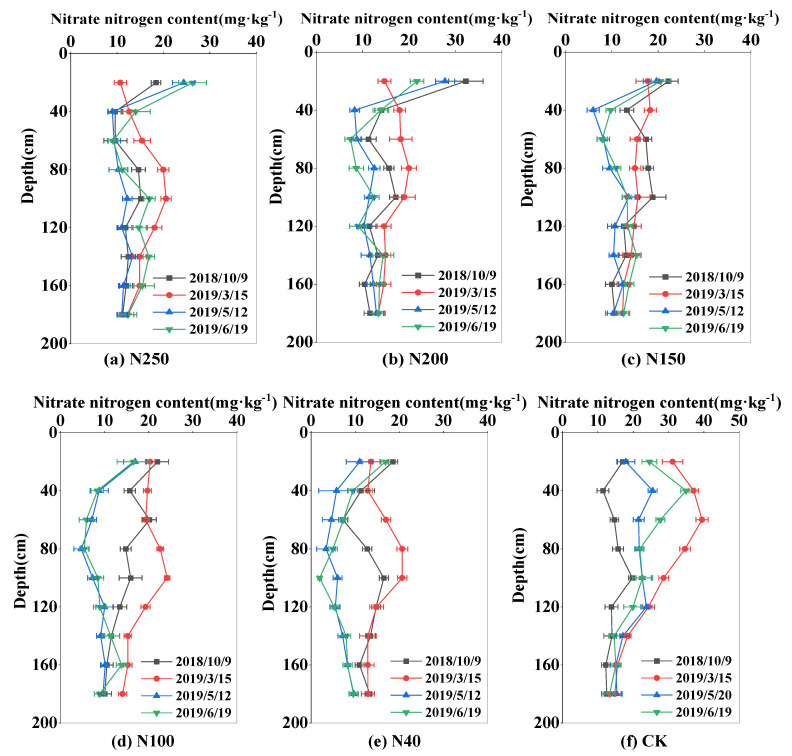
Distribution of nitrate-N content in the 0–180 cm soil layer in the 2018–2019 season. Subfigs (**a**–**f**) represent N treatments of N250, N200, N150, N100, N40, and CK, respectively.

The soil nitrate-N content in the experimental field was high at the beginning of the experiment in October 2017, decreased during the wheat growing period, and reached the lowest level at harvest. At the beginning of the experiment, the nitrate-N content was highest at 60 cm soil depth (42.06 mg·kg^−1^), and the mean value in the 0–100 cm soil layer was 30.43 mg·kg^−1^, with total nitrate-N storage in the 0–100 cm soil layer of 425 kg ha^−1^. During the wheat regreening period in March, the mean nitrate-N content in the 0–100 m soil layer decreased to 18.38 mg·kg^−1^, varied from 17.25 to 28.51 mg·kg^−1^, and reached the lowest level of 8.02–24.83 mg·kg^−1^ at harvest in 2018. A similar pattern of change in the nitrate content was observed in the 2018–2019 season (Figure 5 and Figure 6). The nitrate-N content in the 0–100 cm soil layer showed clear changes, especially in the 0–60 cm soil layer, where most plant roots were located. The soil nitrate-N content was generally lower in the 100–180 cm soil layer than in the 0–100 cm soil layer, and it changed slightly during the whole wheat growing season. The standard deviation of mean nitrate-N content in the 100–180 cm soil layer in the whole wheat growth season was 2.54 mg·kg^−1^ among the five treatments across the two seasons. In the CK, the corresponding standard deviation was 8.36 mg·kg^−1^ in the 0–100 cm soil layer.

The nitrate-N content in the 0–100 cm soil layer was greatly influenced by the N application rate. At wheat harvest, the mean nitrate-N contents in the 0–100 soil layer in N250, N200, N150, N100, N40, and the CK were 17.1, 18.88, 18.59, 15.42, and 12.29 mg·kg^−1^, respectively, in 2018; and 15.51, 12.81, 12.5, 8.94, and 8.09 mg·kg^−1^, respectively, in 2019. The amount of nitrate-N remaining in the soil at wheat harvest increased as the N application rate increased. In addition, the nitrate-N content in the main root zone of the 0–40 cm soil layer at the CK treatment was significantly higher than other N treatments. For the CK, the average nitrate-N content in the 0–40 cm soil layer at wheat harvest was 32 mg·kg^−1^ in 2018 and 29.78 mg·kg^−1^ in 2019, and the other N treatments were 19.1 (N250), 23.76 (N200), 20.41 (N150), 15.47 (N100), and 12.17 (N40) mg·kg^−1^, respectively in 2018 and 20.14 (N250), 17.8 (N200), 15.16 (N150), 12.45 (N100), and 13.15 (N40) mg·kg^−1^ in 2019. This shows that under the local high nitrogen input conditions, wheat cannot fully absorb the applied nitrogen fertilizer, which consequently causes large nitrogen residues.

### 2.4. Winter Wheat Growth Indexes

**Figure 7:**
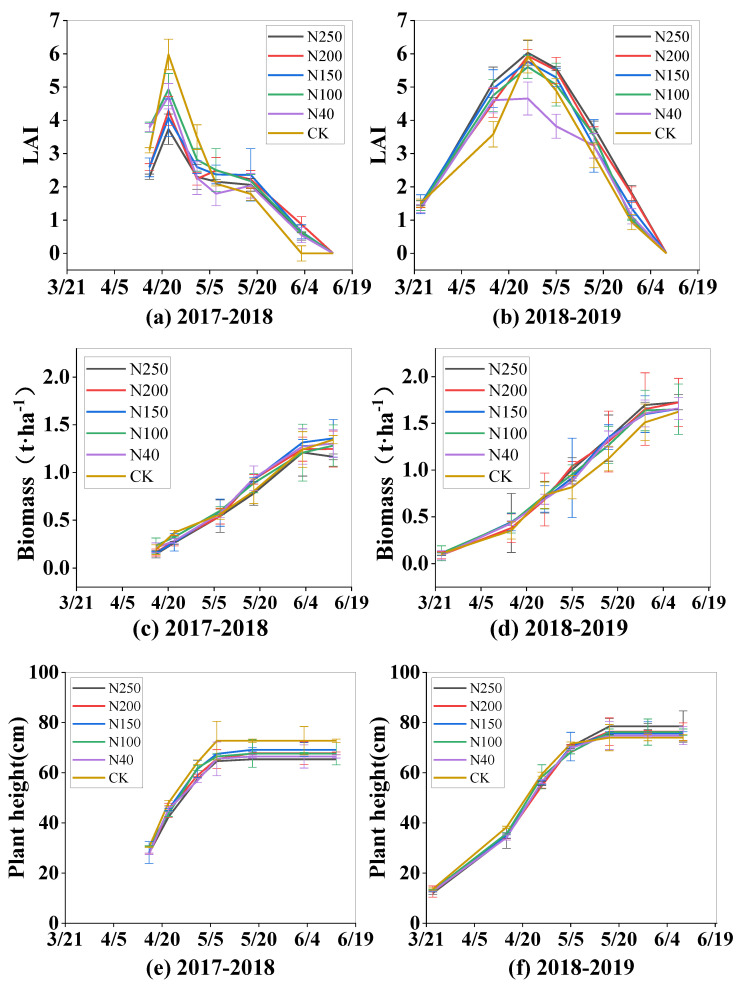

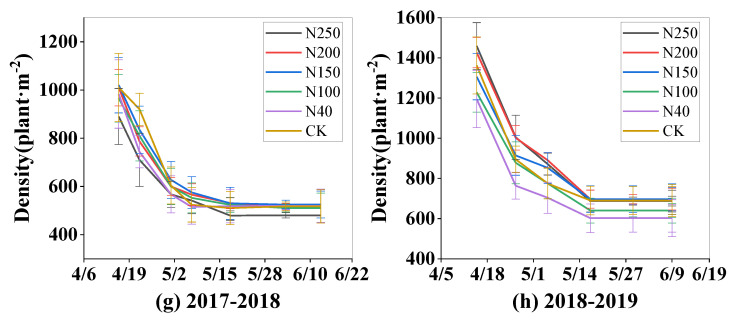
Seasonal patterns of growth indexes (leaf area index (LAI), dry biomass of above-ground plant parts, plant height, plant density) of winter wheat under five N application treatments and the CK in the two experimental seasons. Figures (**a**,**c**,**e**,**g**) show LAI, biomass, plant height, and plant density, respectively, in the 2017–2018 wheat season; and (**b**,**d**,**f**,**h**) show LAI, biomass, plant height, and plant density, respectively, in the 2018–2019 wheat season.

The winter wheat growth indexes, including leaf area index (LAI), dry biomass of above-ground plant parts, plant height, and plant density, are shown in Figure 7. The trends in the changes in the four growth indexes in both seasons were similar among the five treatments and the CK. The four growth indexes were not significantly different among the five treatments (*p* > 0.05) in the first growing season. However, in the second growing season (2018–2019 season), the LAI and plant density were lowest in N40 and were significantly (*p* < 0.05) lower than those in the other four N treatments and the CK during the later growth period. There were significant (*p* < 0.05) differences in the growth indexes between the two seasons. The mean maximum LAI, biomass, plant height, and plant density among the five N treatments were 4.62, 1.28 t·ha^−1^, 67.08 cm, 980 plant·m^−2^, respectively, in the 2017–2018 season, but significantly higher (5.65, 1.67 t·ha^−1^, 75.9 cm, and 1579 plant·m^−2^, respectively) in the 2018–2019 season, showing increases of 22.3%, 30.4%, 13.2%, 61.1%, respectively, in the 2018–2019 season.

### 2.5. Wheat Grain Yield and Harvest Factors

**Figure 8:**
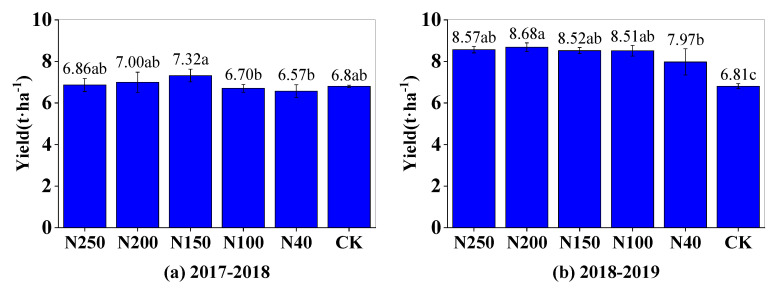
Grain yield of winter wheat under different N application treatments in 2017–2018 (**a**) and 2018–2019 seasons (**b**). Different letters above blue bars indicate significant differences among treatments at 0.05 level (Duncan’s multiple range test).

In the 2017–2018 and 2018–2019 seasons, the highest wheat yield was 7.32 t·ha^−1^ in the N150 treatment and 8.68 t·ha^−1^ in the N200 treatment, respectively (Figure 8). Generally, the wheat yields were not significantly different among the N150, N200, and N250 treatments (*p* > 0.05). However, the yields in those treatments were significantly (*p* < 0.05) higher than those in the N40 treatment and the CK (Figure 8). The wheat yield in the N100 treatment was lower than those in the N150, N200, and N250 treatments, but was generally higher than those in N40 and the CK. Therefore, increasing the N application rate from 40 to 150 kg ha^−1^ greatly improved wheat yield production, but further increases in the N application rate only slightly affected wheat yield. The mean wheat yield among the five N treatments in the 2017–2018 season was 6.89 t·ha^−1^, which was 18.5% lower than that (8.45 t·ha^−1^) in the 2018–2019 season, indicating that there was a substantial variation in wheat yield between the two different growing seasons.

**Figure 9:**
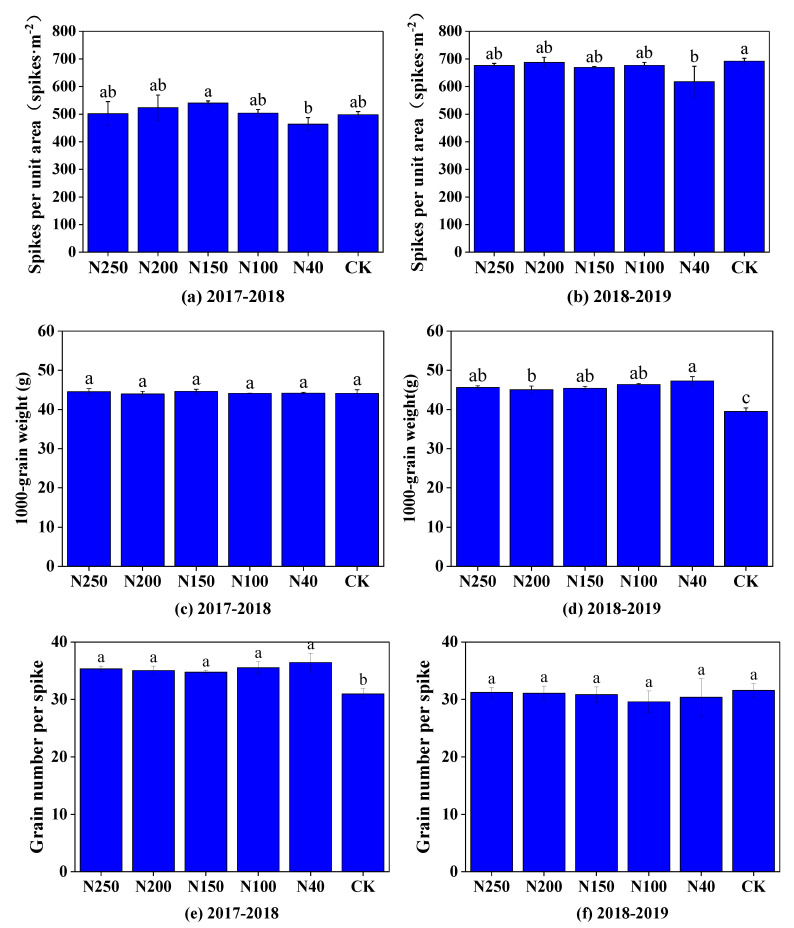
Three harvest-related factors of winter wheat yield (number of spikes per unit area, 1000-grain weight, grain number per spike) under different N application treatments in the two seasons. Figures (**a**,**c**,**e**) show data from the 2017–2018 season, and (**b**,**d**,**f**) show data from the 2018–2019 season. Different letters above blue bars indicate significant differences among treatments at 0.05 level (Duncan’s multiple range test).

The three harvest-related factors, number of spikes per unit area, grain number per spike, and 1000-grain mass, in the two seasons are shown in Figure 9. Generally, the number of spikes per unit area was not significantly (*p* > 0.05) affected by the N application rate when it was higher than 100 kg ha^−1^ but was significantly (*p* < 0.05) reduced when the N application rate was 40 kg ha^−1^ (Figure 9a,b). The grain number per spike and 1000-grain mass were not significantly (*p* > 0.05) affected by the N application rate between 40 and 250 kg ha^−1^ (Figure 9c–f). The three harvest-related factors differed substantially between the two growth seasons. Compared with the 2017–2018 wheat season, in the 2019–2019 growth season, the mean number of spikes per unit area (665 spikes·m^−2^) was 31.4% higher, and the grain number per spike (30.62 grains per spike) was 13.5% lower. The 1000-grain mass was similar in the two seasons: 44.27 g in 2017–2018 and 45.95 g in 2018–2019. 

**Figure 10:**
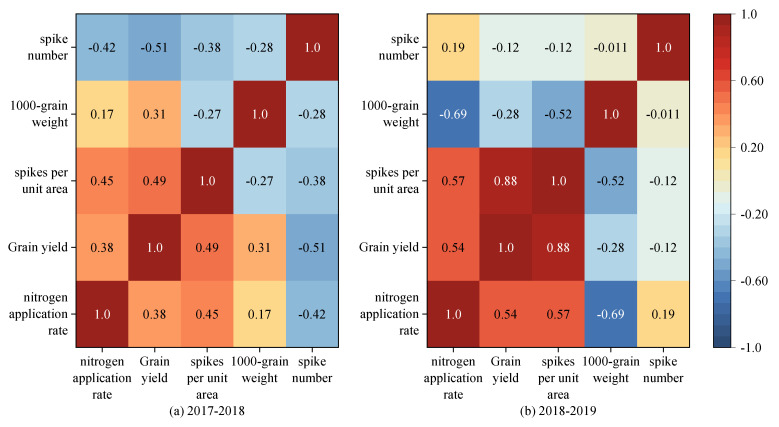
Correlation coefficients between N application rate, winter wheat yield, and the three harvest-related factors in the 2017–2018 (**a**) and 2018–2019 (**b**) growing seasons.

A correlation analysis was conducted to compare the results collected in the two growing seasons (Figure 10). There was a positive correlation between the N application rate and yield, with the correlation coefficients in the two seasons being 0.38 and 0.54 (*p* < 0.05). The number of spikes per unit area was positively and significantly (*p* < 0.05) related to the N application rate and grain yield. The spike number was negatively related to grain yield at the 0.05 level in the 2017–2018 season, but this correlation was not significant in the 2018–2019 season. The 1000-grain weight was negatively correlated with the N application rate at the *p* < 0.01 level in the 2018–2019 season, but this correlation was not significant in the 2017–2018 season. Based on these results, it can be inferred that the N application rate mainly affected the winter wheat yield by affecting the 1000-grain weight and the number of spikes per unit area.

### 2.6. Water- and N-Use Efficiencies

#### 2.6.1. Crop Evapotranspiration, Water- and Fertilizer-Use Efficiency

**Figure 11:**
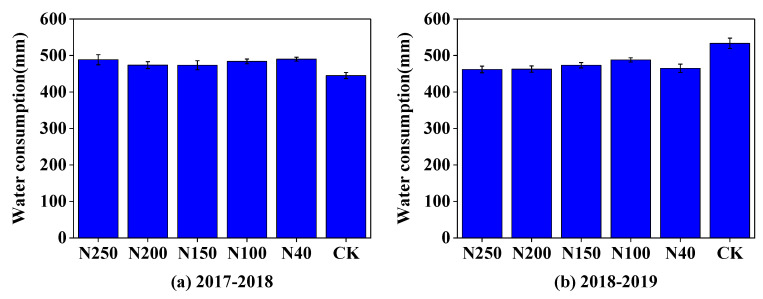
Water consumption of winter wheat plants under different N application treatments during the 2017–2018 (**a**) and 2018–2019 (**b**) growing period.

The average actual crop evapotranspiration (ET_a_) of winter wheat over the five fertilization treatments was 482 mm in the 2017–2018 season and 470 mm in the 2018–2019 season. The ET_a_ values in the CK field in the 2017–2018 and 2018–2019 seasons were 445 and 533 mm, respectively, which were correspondingly 7.7% lower and 13.5% higher than the mean ET_a_ over the five N treatments (Figure 11). Among the five N treatments, the N150 and N200 treatments had the lowest ET_a_ in the 2017–2018 season, and the N200 treatment had the lowest ET_a_ in the 2018–2019 season.

The irrigation water productivity (IWP), crop water productivity (WP), and N-use efficiency for each treatment in the two seasons are listed in Table 1. Among the five N application treatments, N150 had the highest WP and IWP (1.55 and 3.57 kg·m^−3^, respectively) in the 2017–2018 season, and N200 had the highest WP and IWP (1.88 and 2.93 kg·m^−3^, respectively) in the 2018–2019 season. In general, the WP and IWP were not significantly different among N150, N200, and N250, but the WP and IWP of those treatments were significantly higher than those in the treatments with N application rates lower than 100 kg ha^−1^ (Table 1). In both seasons, N40 had the lowest WP and IWP. The WP was higher in all the N application treatments than in the CK. 

The applied N productivity (ANP) ranged from 29.0 to 56.8 kg·kg^−1^ and generally decreased as the N application rate increased. The ANP was highest in the N40 treatment and lowest in the N250 treatment. The ANP in the CK was 25.7 and 26.4 kg·kg^−1^ in the 2017–2018 and 2018–2019 seasons, respectively, lower than the ANP in the five N application treatments in each corresponding season. 

The values of the three indicators (WP, IWP, and ANP) were lower in the 2017–2018 season than in the 2018–2019 season. The values of IWP, WP, and ANP in the 2018–2019 season were 46.9–60.1%, 34.3–46.8%, and 2.5–18.0% higher than their corresponding values in the 2017–2018 season.

#### 2.6.2. Crop N Uptake and Soil N Balance

**Figure 12:**
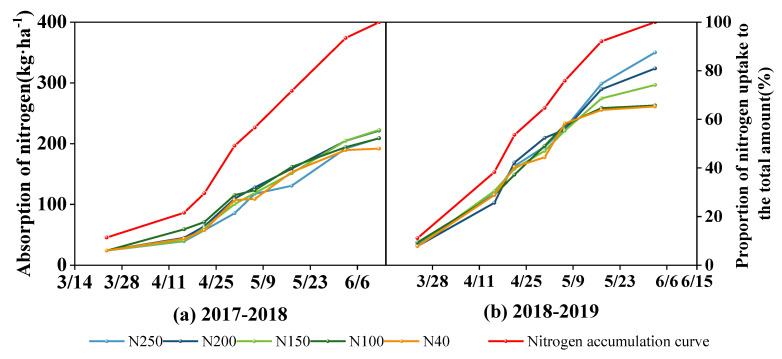
Nitrogen accumulation process and percentage change (the percentage of the average nitrogen absorption of the five treatments to the total average absorption) in above-ground biomass after wheat regreening in the 2017–2018 (**a**) and 2018–2019 seasons (**b**).

Figure 12 shows the curves of crop N uptake by biomass above the ground surface and its proportion of total N uptake during the period from regreening to harvest in the two experimental seasons. At the regreening stage, the N uptake of the wheat crop was approximately 12% of the total N uptake in the whole growth period. During the period when winter wheat grew slowly between the end of the regreening stage and the beginning of the elongation stage, the N uptake reached 22% of the seasonal total. From the elongation stage to the middle of the grain-filling stage, the wheat plants grew quickly and the N uptake amount linearly increased. The proportion of N uptake out of the total seasonal total N uptake was 49% by the flowering stage and 72% and 93% by the early and late grain-filling stages, respectively. From the late grain-filling stage to harvest, the N uptake increased slightly, and the total N uptake amount during this period was approximately 7% of the seasonal N uptake amount. 

The mean seasonal N uptake of winter wheat in the 2017–2018 and 2018–2019 seasons was 210 and 299 kg ha^−1^, respectively, and the corresponding N content in the grains at harvest was 140 and 195 kg ha^−1^, respectively, accounting for 66.7% and 65.22% of the plant total N uptake in the two seasons, respectively. The N uptake in the first season did not differ significantly among the five treatments (*p* > 0.05). However, in the 2018–2019 season, the highest N uptake amount was 350 kg ha^−1^ in the N250 treatment, followed by 323 kg ha^−1^ (N200), 296 kg ha^−1^ (N150), 262 kg ha^−1^ (N100), and 261 kg ha^−1^ (N40). 

The average initial soil nitrate-N content in the 0–200 cm soil layer was 566 kg ha^−1^ pre-plant and 358 kg ha^−1^ at harvest in 2017–2018, and 403 kg ha^−1^ pre-plant and 326 kg ha^−1^ at harvest in 2018–2019. This indicates that after two wheat growing seasons, the soil nitrate-N content in the experimental area was effectively reduced (Table 2). The total N content in the above-ground biomass at harvest in the N250, N200, N150, N100, and N40 treatments was 209, 221, 222, 209, and 192 kg ha^−1^, respectively, in 2017–2018, and 350, 324, 297, 263, and 261 kg ha^−1^, respectively, in 2018–2019. The total N uptake by wheat was higher in 2018–2019 (299 kg ha^−1^) than in 2017–2018 (211 kg ha^−1^). Generally, the amount of N taken up by wheat increased as the N application rate increased. The overall apparent N loss was substantially lower in 2018–2019 (13 kg ha^−1^) than in 2017–2018 (153 kg ha^−1^).

## 3. Discussion

### 3.1. Distribution of Soil Water and Nitrate-N under Different N Application Levels by Sprinkler Irrigation

In this study, with the optimal irrigation scheme, the soil water content in the experimental field mainly changed in the 0–100 cm soil layer, and that in the 100–180 cm soil layer remained relatively stable. This shows that winter wheat under sprinkler irrigation mainly used the soil water in the 0–100 cm soil layer. Approximately 80% of wheat roots are distributed in the 0–80 cm soil layer [20], and water uptake by roots may result in obvious variations in soil water content in the upper 100 cm soil layer. However, in the CK field with local traditional irrigation management, the soil water content showed marked fluctuations below 100 cm soil depth, indicative of deep water seepage. The comparison of patterns in soil water content between optimized sprinkler irrigation and local water management shows that the former system results in good soil water conditions in the root zone and reduces water seepage. Similar results have been reported in other studies [21,22] 

The distribution and transport of nitrate-N in soil are strongly affected by different N application regimes and by irrigation management. Because nitrate-N generally moves along with water flow in soil, there are great variations in soil nitrate-N in the 0–120 cm soil layer. Our results show that winter wheat in the experimental area with optimized sprinkler irrigation management mainly took up nitrate-N in the 0–120 cm soil layer (Figure 5 and Figure 6). Under traditional water and fertilizer management, i.e., high water and high fertilizer inputs, the amount of nitrate-N stored in the CK field was higher than that in the N treatments. Liu et al. [23] investigated nitrate-N distribution and movement in wheat–maize and vegetable fields and found that high irrigation (260–300 mm) and N application (1000 kg ha^−1^) for vegetable cultivation in both open fields and greenhouses resulted in a nitrate-N concentration of 10–15 mg kg^−1^ in the 2–6 m soil layer, which was up to 100% more than that (6–8 mg kg^−1^) in the wheat–maize field with optimized N (400 kg ha^−1^) and water (240 mm) applications under sprinkler irrigation. Li et al. [24] also reported that after a 4-year wheat–maize field experiment under optimal sprinkler irrigation, the applied water and fertilizer N mostly accumulated in the 0–60 cm soil layer in the winter wheat season, and the amount of nitrate-N leaching out of the root zone (0–100 cm) was negligible.

### 3.2. Relationship between Wheat Grain Yield and N Application

Nitrogen, as one of the essential elements required for wheat growth, plays an important role in the growth and yield production of wheat [25]. Studies have shown that an appropriate increase in the amount of N fertilizer can improve the photosynthesis rate of wheat, promote the accumulation of above-ground dry matter mass, and thus increase wheat yield [5,26]. In this study, when the N application rate was lower than 200 kg ha^−1^, yield and the three yield-related factors (number of spikes per unit area, grain number per spike, and 1000-grain mass) generally increased as the N application rate increased (Figure 8 and Figure 9). The correlation analysis between the N application rate and the three factors related to wheat yield showed that increases in the 1000-grain mass and the number of spikes per unit area were the main reasons for the increase in wheat grain yield under higher N application rates. This result is consistent with those of previous studies [27,28,29,30]. Considering the insignificant difference in wheat yield between the N150 and N200 treatments, the optimal N application range was 150–200 kg ha^−1^.

The NCP produces approximately 60% of the total wheat crop in China [7]. It has been reported that the wheat yield in the NCP increased from 6000 kg ha^−1^ in the 1990s to 7000 kg ha^−1^ in the 2000s, and then to 8500 kg ha^−1^ in the 2010s (Table 3). Similarly, the regional mean wheat yield, as listed in the national statistical database, increased from 3738 kg ha^−1^ in the 1990s to 4739 kg ha^−1^ in the 2000s and then 5630 kg ha^−1^ in the 2010s [7,31]. During the same period (from the 1990s to 2010s), the recommended optimized N application rate only changed slightly, from 150–210 kg ha^−1^ to 180–220 kg ha^−1^ (Table 3). The mean wheat yields in the last 30 years, as reported in the published literature and the national statistical database, have increased by 41.7% and 50.6%, respectively, while the mean optimized N application reported in the literature has ranged from 150 to 200 kg ha^−1^ (Table 3). This means that the applied N productivity of wheat in the NCP has greatly improved. This may be because of new wheat varieties being cultivated, improved field pest and weed control, and optimization of water and fertilizer management [32]. 

Compared with traditional surface irrigation, the sprinkler irrigation method distributes water more uniformly and precisely, establishes the optimal soil water content for crop growth, and can regulate the microclimate in the field to enhance leaf photosynthetic activity and mitigate the dry-hot-windy climate [33]. In this study, the optimized N application rate was 150–200 kg ha^−1^, and sprinkler irrigation was able to distribute water and N to the main root zone in the 0–60 cm soil layer (Figure 3, Figure 4, Figure 5 and Figure 6), which improved wheat growth and grain yields (Figure 7 and Figure 8). Therefore, the change from the traditional surface irrigation system to the sprinkler fertigation system could be the main reason for the increases in wheat yield and water- and N-use efficiencies in the last 30 years [34].

**Table 3:** Optimized N fertilization rates, wheat grain yields, and irrigation systems in the 1990s, 2000s, and 2010s.

Period	Optimized N Rate (kg ha^−1^)	Wheat Grain Yield (kg ha^−1^)	Irrigation Depth and System (mm)	References
1990s	180–220	5250–6000	180–200 Border irrigation	[18,35,36,37,38]
2000s	155–210	6500–7000	180–270 Border irrigation	[10,17,39,40,41,42,43,44,45]
2010s	150–210	7500–8500	180–230 Drip irrigation or sprinkler irrigation	[19,46,47,48,49,50,51,52]
2017–2019	150–200	7300–8500	200–250 Sprinkler irrigation	this study

### 3.3. Soil N Balance and N-Use Efficiency under Different N Application Rates with Sprinkler Fertigation

Under appropriate soil water conditions, optimal fertilizer application can improve the soil N status in the crop root zone and enhance the N-use efficiency [53]. In another study on sprinkler fertigation in an experimental wheat field, the contents of soil water and nitrate-N greatly varied in the 0–60 cm soil layer, and no water and N seepage were detected below 100 cm soil depth [21]. Similar results were obtained in this study (Figure 3, Figure 4, Figure 5 and Figure 6). However, high levels of N and water application to a vegetable field caused nitrate-N to accumulate at 200.2% higher levels in the 0–100 cm soil layer compared with that in a wheat–maize rotation cultivation system [54]. Ultimately, high levels of nitrate-N in the 0–100 cm soil layer can lead to high N leaching and groundwater pollution [55]. The sprinkler fertigation system, accompanied by optimal sprinkler irrigation scheduling, more effectively distributes water and N in the root zone, ultimately reducing N leaching and increasing the N-use efficiency [56,57,58]. 

The N balance in the soil is greatly affected by the amount of N applied, the amount of N taken up by crops, and crops’ N-use efficiency [25,53]. Li et al. [24] investigated the accumulation of N in a wheat–maize system after a 4-year field experiment in the NCP and reported that the nitrate-N concentration in 0–100 cm soil layer varied slightly in the 4th year; nitrate-N leaching was negligible when N was applied at rates lower than 220 kg ha^−1^ yr^−1^; and the amount of nitrate-N leaching was linearly related to the amount of N applied within the range of 400–600 kg ha^−1^ yr^−1^. In this study, the amount of nitrate-N in the soil in the root zone (the 0–100 cm soil layer) before wheat sowing decreased from 566 kg ha^−1^ in the first season to 403 kg ha^−1^ in the second season and apparent N loss from the soil during the whole wheat season decreased from 153 kg ha^−1^ in the 2017–2018 season to 13 kg ha^−1^ in the 2018–2019 season. These results show that optimal N application scheduling with the sprinkler fertigation system was helpful to reduce N losses without reducing wheat yield and ultimately improved the N-use efficiency of winter wheat. Balanced N conditions in the root zone could provide sustainable soil fertility for crop production. With the aim of maintaining the soil N balance and sustainable wheat production, the optimal amount of N applied during the winter wheat season is recommended at 200 kg ha^−1^ to achieve a wheat yield of approximately 8–9 t ha^−1^.

## 4. Materials and Methods

### 4.1. Experimental Site

This field experiment was conducted during the 2017–2018 and 2018–2019 winter wheat growing seasons at the National Seed Breeding Station (NSBS) in Ningjin County, Xingtai City, Hebei Province (37°29′, 114°55′ E, 26.4 m altitude), China. The station is located in the central NCP. The NCP has a continental monsoon climate, with an annual average temperature of 12.8 °C and an average frost-free period of approximately 200 days. The annual average precipitation is between 452 and 553 mm, approximately 60%-70% of which is concentrated in summer from July to September. The annual average sunshine duration is 2538 h, and the wind speed range is 2.3–2.7 m·s^−1^.

The proportions of clay, silt, and sand particles in soil at the experimental station were 10–7%, 51–67%, and 19–35%, respectively. The soil texture in the 0–200 cm soil layer was silt loam and soil clay following the American classification. The field soil water holding capacity ranged from 0.36 to 0.38 cm^3^·cm^−3,^ and the soil bulk density ranged from 1.35 to 1.77 g·cm^−3^ (Table 4).

### 4.2. Experimental Design

#### 4.2.1. N application Treatments

According to our investigations, the range of total N, phosphorus (P), and potassium (K) fertilizers applied in the winter wheat growing season under the local traditional practice are 250–300, 45–65, and 50–85 kg ha^−1^, respectively. The fertilizer used for base fertilizer is compound fertilizer (N, 18%; P, 5%; K, 4%). Under the local practice, all P and K fertilizers are applied pre-plant as basal fertilizers. Approximately half of the N fertilizer is applied as basal fertilizer, and the other half is applied once or twice after wheat regreening as topdressing fertilizer (using urea with N content of 46%). The total irrigation water in the wheat season is approximately 300 mm, of which approximately 100 mm is applied in November as over-winter irrigation, 100 mm after regreening in March, and 100 mm at the heading and grain-filling stages in late April and May. If the soil water content is so low that seed emergence is inhibited, approximately 60–80 mm of water is applied to promote the emergence of winter wheat.

Based on the local N application amount of 250–300 kg ha^−1^ during the whole winter wheat season, five N application treatments were established in this study: 250, 200, 150, 100, and 40 kg ha^−1^, designated as N250, N200, N150, N100, and N40, respectively. The ratio of topdressing N fertilizer applied at the regreening, jointing, and grain-filling stages was 1:1:1. To ensure the growth of winter wheat at the seedling stage and overwintering stage, the amount of basal N fertilizer applied in all treatments was 40 kg ha^−1^. Therefore, no topdressing N was applied in treatment N40. For the treatments N250, N200, N150, and N100, the remaining N amounts of 210, 160, 110, and 60 kg ha^−1^ were applied as topdressing in three equal amounts at the regreening, jointing, and grain-filling stages. All five N treatments were replicated three times. A wheat field with local traditional water and fertilizer management, which was located on the east side of the experimental field, served as the control (CK). Via observation, the wheat growth stages and the corresponding period in the two experimental seasons are shown in Table 5. The specific N application rates at different growth stages in each treatment are listed in Table 6.

**Figure 13:**
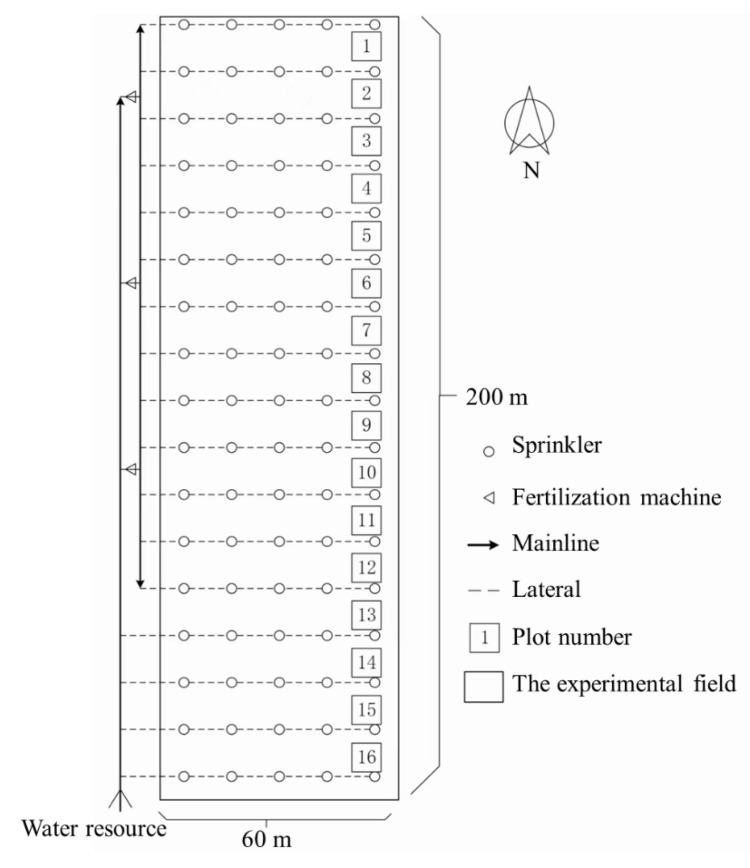
The specific layout of the experiment.

The total experimental field was 60 m wide × 200 m long. Along the long side in the South–North direction were 16 plots, each with a size of 12 m × 60 m. Among the 16 plots, plots 1–3 were used for the N250, plots 4–6 were used for the N200, plots 7–9 were used for the N150, plots 10–12 were used for the N100 treatment, and plots 13–16 were used for the N40 treatment (Figure 13). The experimental field was irrigated using a sprinkler irrigation system. Each plot included four subplots measuring 12 m × 12 m each. Regulated angle sprinklers (model PYTK 10, Wudayunshui Co., Ltd., Wuhan, China) were located on 1 m high risers on the sub-laterals at intervals of 12 m. The sprinkler discharge rate was 0.9 m^3^ h^−1^ with a spraying radius of approximately 12 m under normal working pressure of 0.25 MPa. Three sets of fertilizer-injecting machines were located between the mainline and laterals to apply topdressing N fertilizer (urea). Irrigation water was pumped from groundwater with the water level approximately 40 m below the ground surface.

The winter wheat cultivar ‘*YingBo-700*,’ which is recommended by the local government, was sown at a density of approximately 4.5 million seeds with 20 cm row spacing on 24 October 2017 and 17 October 2018. The crops were harvested on 13 June 2018 and 9 June 2019. Other field management activities, including weed and insect control, were conducted in the same way as in the other fields at the experimental station.

#### 4.2.2. Irrigation Management

Based on the research results of our group, irrigation was first applied to a depth of approximately 40 mm after wheat regreening in the middle of March. After that, the irrigation depth was calculated as 0.65 times the difference between the cumulative water surface evaporation from a 20 cm pan placed on the top of the canopy and rainfall between two irrigation intervals. Generally, the time interval between two irrigation events was 10–14 days, and the irrigation depth was 30–40 mm per event [16]. Topdressing N was applied to the field with sprinkler irrigation at set growth stages of winter wheat. The total irrigation depth in the five N treatments in the 2017–2018 and 2018–2019 winter wheat seasons was 204.9 mm and 296.2 mm, respectively; and the total irrigation depth in the CK in the two corresponding seasons was 191 mm and 372.3 mm, respectively. The specific amount of irrigation is shown in Table 7.

### 4.3. Measurements and Methods

#### 4.3.1. Soil Water Content and Nitrate-N Content

Soil samples were collected at 20 cm intervals in the 0–180 cm soil layer for soil water content and nitrate-N content measurements pre-plant and at the regreening, jointing, and grouting stage. At each measurement, soil samples were collected at three sites for soil water and nitrate-N content measurements in each treatment, and the mean value of the three replicates was used for analysis. The soil water content was measured by the oven-drying method. The specific method is soil samples were collected by auger, stored in aluminum boxes, and weighed using a 0.1 g precision balance (wet weight). The soil samples were then placed in an oven (105 °C) and dried for 6–8 h until constant weight (dry weight). Next, the water in the soil sample was calculated as the difference between the wet-weight and dry-weight of the soil sample. Lastly, the gravity soil water content was calculated as the ratio of the soil water to the dry soil mass. The volumetric soil water content was calculated as the product of gravity soil water content and the soil bulk density (Table 4).

For soil nitrate-N content measurement, the soil samples were first air-dried and then milled and screened through a 2 mm sieve. The 10 g soil samples were put into a container, adding 50 mL 2 mol/L KCl solution (soil/solution: 1:5) in it, and the mixture was shaken for 1 h on a rotary shaker (220 rev·min-1) and filtered. After adding 1 mL 1 mol/L HCl solution to the filtered extract, the mixed solution was directly analyzed to determine the concentration of nitrate-N by ultraviolet spectrophotometry (Shanghai Mapada Instruments Co. Ltd., Shanghai, China) using a dual-wavelength method (220 nm, 275 nm).

#### 4.3.2. Wheat Growth Indexes

The wheat growth indexes measured in this study included crop height, plant density, leaf area index (LAI), and dry biomass of above-ground plant parts. Three 1 m rows of wheat in each treatment were selected, and the number of wheat plants was counted. Then, the plant density was calculated from the mean number of wheat per 1 m row with a row spacing of 0.2 m. In each treatment, three clusters of wheat with approximately 60 stems were cut at the base of the stem, all green leaves were picked, and the leaf area was measured using a leaf area instrument (model LI-3000C, LI-COR Inc., Lincoln, NE, USA). Then, the LAI was calculated from the mean leaf area per stem and plant density. The stems and all leaves collected in each treatment were dried at 105 °C for 30 min and then at 70–80 °C for 24 h to constant weight. Then, the mass of stems and leaves was measured using a 0.01 g resolution balance. These values were converted to per-hectare values based on plant density. The wheat growth indexes were investigated approximately every 2 weeks. The plant height and density were not measured after heading because they did not change after this stage.

#### 4.3.3. Grain Yield and Harvest Factors

At harvest, all wheat plants were harvested from ten 1 m × 1 m subplots in each treatment for analyses of yield- and harvest-related factors. For each sample, wheat grains were obtained by threshing, and then the water content of the wheat grains was determined. The final grain yield of each treatment was obtained based on the standard water content of 12.5%. In each treatment, 20 wheat plants were selected to count the number of kernels per spike and the number of spikes. The 1000-grain weight was determined for 10 separate samples of the grains collected for yield measurement from each treatment.

#### 4.3.4. Meteorological Indexes

Data for meteorological variables, including air temperature, air humidity, radiation, wind speed, soil profile temperature, soil surface heat flux, and rainfall, were obtained from an automatic weather station deployed at the experimental station. All data were sampled at 10 s intervals, and 30 min mean data were stored by a CR1000 data logger (Campbell Scientific, Inc., Logan, UT, USA). The water surface evaporation from the 20 cm pan (Model ADM7, Zhonghuantianyi Co., Ltd., Tianjin, China) above the wheat canopy was measured daily at approximately 8:00 before sunrise.

### 4.4. Calculations

#### 4.4.1. Crop water consumption

The soil water balance method was used to calculate crop evapotranspiration (*ET_a_*) during the whole growth period of winter wheat. The formula is defined as follows:

*ET_a_ = (P_0_ + I + W) − (R_0_ + D +* Δ*S)* (1)

where *ET_a_* is the actual crop water consumption during a period of time (mm), *P_0_* is the effective rainfall (mm), *I* is the irrigation depth (mm), *W* is groundwater recharge (mm), *R_0_* is the surface runoff (mm), *D* is the deep leakage (mm), and *S* is the variation in soil water storage (mm). In this study, the groundwater level was approximately 40 m lower than the ground surface, the field was flat, and irrigation water was well controlled. Therefore, the flux of *W*, *R_o,_* and *D* were taken as zero during the calculation of *ET_a_* using Equation (1).

#### 4.4.2. Water- and N-Use Efficiencies

Water productivity, irrigation water productivity, and N productivity were calculated using Equations (2)–(4), respectively, as follows:

(2) $$\mathrm{WP}=\frac{0.1Y}{ET_{a}}$$

(3) $$\mathrm{IWP}=\frac{0.1Y}{I}$$

(4) $$\mathrm{ANP}=\frac{Y}{N}\;\times \;100\%$$

where *WP* is water productivity (kg·m^−3^), *IWP* is irrigation water productivity (kg·m^−3^), *ET_a_* is the actual crop water consumption (mm), *I* is the effective irrigation amount (mm), *Y* is grain yield (kg ha^−1^), *ANP* is the applied N productivity, and *N* is the N application rate (kg ha^−1^).

#### 4.4.3. Nitrogen Balance

Because the content of ammonium-N in local soil was low and the change was small [59], it was ignored in these calculations. The following formula was used to calculate the soil N balance:

(5) $$N_{0}(N_{\mathrm{final}})\;\;\frac{Z\times \rho \times C_{NO_{3}-N}}{10}$$

(6) $$N_{\mathrm{crop}}=\frac{M_{d}\times C_{crop}}{1000}$$

*N_m_ = N_crop_ + N_0_* − *N_final_* − *N_ir_* (7)

*N_loss_ = N_0_ + N_fertilizer_
+ N_m_ + N_ir_* − *N_final_* − *N_crop_* (8)

where *N*_0_ is the initial soil nitrate-N content (kg ha^−1^), *N_final_* is the soil nitrate-N content at harvest (kg ha^−1^), *Z* is the soil depth (cm), *ρ* is soil bulk density (g cm^−3^), *C_NO3-N_* is the concentration of N0_3_-N in soil (kg ha^−1^), *N_crop_* is the amount of N absorbed by above-ground crops (kg ha^−1^), *M_d_* is the dry above-ground biomass of crops (kg ha^−1^), *C_crop_* is the total N in the above-ground biomass at harvest (g kg^−1^), *N_m_* is the amount of N mineralization in the CK (kg ha^−1^), *N_ir_* is the amount of N supplied by irrigation (kg ha^−1^), *N_loss_* is the apparent N loss (kg ha^−1^), and *N_fertilizer_* is the amount of N applied as fertilizer (kg ha^−1^).

### 4.5. Statistical Analyses

Microsoft Excel 2019 was used to calculate and process the data obtained in the field. Analysis of variance was performed using the original model procedure of Origin2021 (OriginLab Corporation, Northampton, MA, USA) to test the effects of different N treatments on yield and water- and fertilizer-use efficiencies. Differences were determined using Duncan’s multiple range test at the 5% level of significance. Pearson’s correlation analysis was conducted to analyze the correlation between the N application rate and yield.

## 5. Conclusions

A two-winter-wheat field experiment with five N application rates was conducted in the NCP, and results showed that under optimized sprinkler irrigation conditions, the applied water and nitrogen (nitrate nitrogen) content were mainly distributed in the 0–100 cm soil layer. Nitrogen application range of 150–200 kg ha^−1^ significantly (*p* < 0.05) improved the agronomic traits of crops and increased the number of spikes per unit area and 1000-grain mass, thereby producing the highest wheat yield and enhanced nitrogen use efficiency. Higher N applications from 200 to 250 kg ha^−1^ did not show higher benefits in yield and nitrogen use efficiency. Considering sustainable field development and higher N-use efficiency, an optimal N application rate of 200 kg ha^−1^ is recommended for high winter wheat production under sprinkler fertigation conditions in the NCP.

## Figures and Tables

**Table 1:** Effects of different N application rates on water productivity (WP), irrigation water productivity (IWP), and applied N productivity (ANP) of winter wheat crops.

Treatment	2017–2018			2018–2019		
	WP (kg·m^−3^)	IWP (kg·m^−3^)	ANP (kg·kg^−1^)	WP (kg·m^−3^)	IWP (kg·m^−3^)	ANP (kg·kg^−1^)
N250	1.41 bc	3.35 c	29.05 e	1.86 a	2.89 a	34.27 e
N200	1.48 ab	3.42 b	37.03 d	1.88 a	2.93 a	43.41 d
N150	1.55 a	3.57 a	51.62 c	1.80 b	2.88 a	56.78 c
N100	1.39 c	3.27 d	70.94 b	1.74 c	2.87 a	85.11 b
N40	1.34 c	3.2 e	178.22 a	1.72 c	2.69 a	199.37 a
CK	1.63 a	3.79 a	25.76 e	1.28 d	1.83 b	26.39 f
Average	1.41	3.36	47.20	1.86	2.85	54.89

Only N250-N40 test treatments were included in the average value calculation. Different letters in the same column indicate significant differences among treatments at the 0.05 level.

**Table 2:** Nitrogen balance during the 2017–2018 and 2018–2019 growing seasons of winter wheat.

Seasons		N Balance	N250	N200	N150	N100	N40
2017–2018	N input	Initial nitrogen content	641	520	619	564	485
		Nitrogen fertilizer amount	238	191	144	97	40
		Irrigation water nitrogen	14	14	14	14	14
		Mineralized nitrogen	0	0	0	0	0
	N output	Nitrogen absorption	209	221	222	209	192
		Residual nitrogen amount	397	390	389	337	276
		Apparent nitrogen loss	286	113	166	129	72
2018–2019	N input	Initial nitrogen content	414	425	428	404	346
		Nitrogen fertilizer amount	250	200	150	100	40
		Irrigation water nitrogen	18	18	18	18	18
		Mineralized nitrogen	45	45	45	45	45
	N output	Nitrogen absorption	350	324	297	263	261
		Residual nitrogen amount	484	371	348	237	187
		Apparent nitrogen loss	0	0	0	66	0

**Table 4:** Soil particle distribution, soil texture, and bulk density in the 0–200 cm soil layer.

Depth (cm)	Soil Particle Distribution (%)			Soil Texture *	Bulk Density
	Clay	Silt	Sand		g·cm^−3^
0–20	9.7	61.2	29.1	Silty loam	1.35
20–40	9.8	61.8	28.4	Silty loam	1.39
40–60	13.9	67.0	19.2	Silty loam	1.44
60–80	16.9	54.7	28.5	Silty loam	1.47
80–100	14.5	51.0	34.6	Silty loam	1.58
100–120	13.3	67.6	19.1	Silty loam	1.77
120–140	12.4	61.1	26.5	Silty loam	1.75
140–160	10.7	56.0	33.3	Silty loam	1.70
160–180	11.1	57.7	31.2	Silty loam	1.68
180–200	11.8	61.9	26.3	Silty loam	1.69

Note: * Soil texture is classified based on the American soil texture standard.

**Table 5:** The definition of each growth stage and the corresponding growth period in the two experimental seasons.

Wheat Seasons	Wheat Growth Stages and Corresponding Period						
	Sowing Period (SP)	Seedling Stage (ST)	Wintering Stage (WT)	Regreening Stage (RT)	Jointing and Flowering Stage (JT)	Grouting Stage (GT)	Maturing Stage (MT)
2017–2018	24 October 2017	25 October–20 November	21 November–17 February	18 February–5 April	6 April–5 May	6–31 May	1–13 June
2018–2019	17 October 2018	18 October–20 November	21 November–11 February	12 February–2 April	3 April–1 May	2–31 May	1–9 June

**Table 6:** Planned N application rates at different growth stages in each treatment (kg ha^−1^).

Treatments	Total N Fertilizer Amount	Base Nitrogen	Topdressing Nitrogen		
		At Sowing	Regreening Stage	Jointing and Flowering Stage	Grouting Stage
N250	250	40	70	70	70
N200	200	40	53	53	53
N150	150	40	37	37	37
N100	100	40	20	20	20
N40	40	40	0	0	0
CK	250	110	70	70	0

**Table 7:** Irrigation time and depth in the five N treatments under sprinkler fertigated field and in the control field in the two experimental seasons.

Wheat Seasons	Growth Stage	Nitrogen Treatments		Control Field (CK)	
		Date	Irrigation Depth (mm)	Date	Irrigation Depth (mm)
2017–2018	SP *	15 November 2017	80	15 November 2017	80
	RT	1 April 2018	41.6	27 March 2018	111
	JT	16 April 2018	34.7		
	GT	8 May 2018	20.8		
		25 May 2018	27.8		
		Total	204.9	Total	191
2018–2019	SP	18 October 2018	67.8	17 October 2018	71
	WT	4 December 2018	94.5	4 December 2018	94.5
	RT	29 March 2019	36.2	27 March 2019	107.8
	JT	20 April 2019	8.9	27 April 2019	99
		23 April 2019	26.4		
	GT	8 May 2019	37.4		
		21 May 2019	25		
		Total	296.2	Total	372.3

Note: * The abbreviated letters “SP”, “WT”, “RT”, “JT”, and “GT ” represent the wheat growth stage of the sowing period, wintering stage, regreening stage, jointing stage, and grouting stage, respectively. The definition of growth stage can be found in Table 5.

## Data Availability

Data will be available after we evaluate the requirement.
